# Functional properties of insect olfactory receptors: ionotropic receptors and odorant receptors

**DOI:** 10.1007/s00441-020-03363-x

**Published:** 2021-01-27

**Authors:** Dieter Wicher, Fabio Miazzi

**Affiliations:** 1 Department of Evolutionary Neuroethology, Max Planck Institute for Chemical Ecology, Hans-Knoell-Str. 8, 07745 Jena, Germany; 2 Research Group Predators and Toxic Prey, Max Planck Institute for Chemical Ecology, Hans-Knoell-Str. 8, 07745 Jena, Germany

**Keywords:** Insect olfaction, Ionotropic receptor, Odorant receptor, Ion channel, Olfactory sensory neuron, Signal transduction, Sensitization, Adaptation

## Abstract

The majority of insect olfactory receptors belong to two distinct protein families, the ionotropic receptors (IRs), which are related to the ionotropic glutamate receptor family, and the odorant receptors (ORs), which evolved from the gustatory receptor family. Both receptor types assemble to heteromeric ligand-gated cation channels composed of odor-specific receptor proteins and co-receptor proteins. We here present in short the current view on evolution, function, and regulation of IRs and ORs. Special attention is given on how their functional properties can meet the environmental and ecological challenges an insect has to face.

## Introduction

The olfactory system is dedicated to detect and to encode information from volatile chemical signals. Such signals can be categorized according to the information they transfer. For example, chemosignals involved in social communication may be informative solely for the receiver as an olfactory cue, or they may carry useful information for both the emitter and the receiver as semiochemicals (Hansson and Wicher 2016). Semiochemicals emitted and perceived within a species act as pheromones while interspecific signals are allelochemicals. The latter may be beneficial for the receiver as kairomones or for the emitter as allomones. For insects the sense of smell is essential for central tasks such as to find food sources or mating partners or to avoid life-threatening events.

The insect olfactory receptors belong to two families, the odorant receptors (ORs), and the so-called ionotropic receptors (IRs). The first members of the OR family were discovered two decades ago (Clyne et al. 1999; Gao and Chess 1999; Vosshall et al. 1999), whereas the IRs that are related to ionotropic glutamate receptors were first reported ten years later (Benton et al. 2009).

**Fig. 1:**
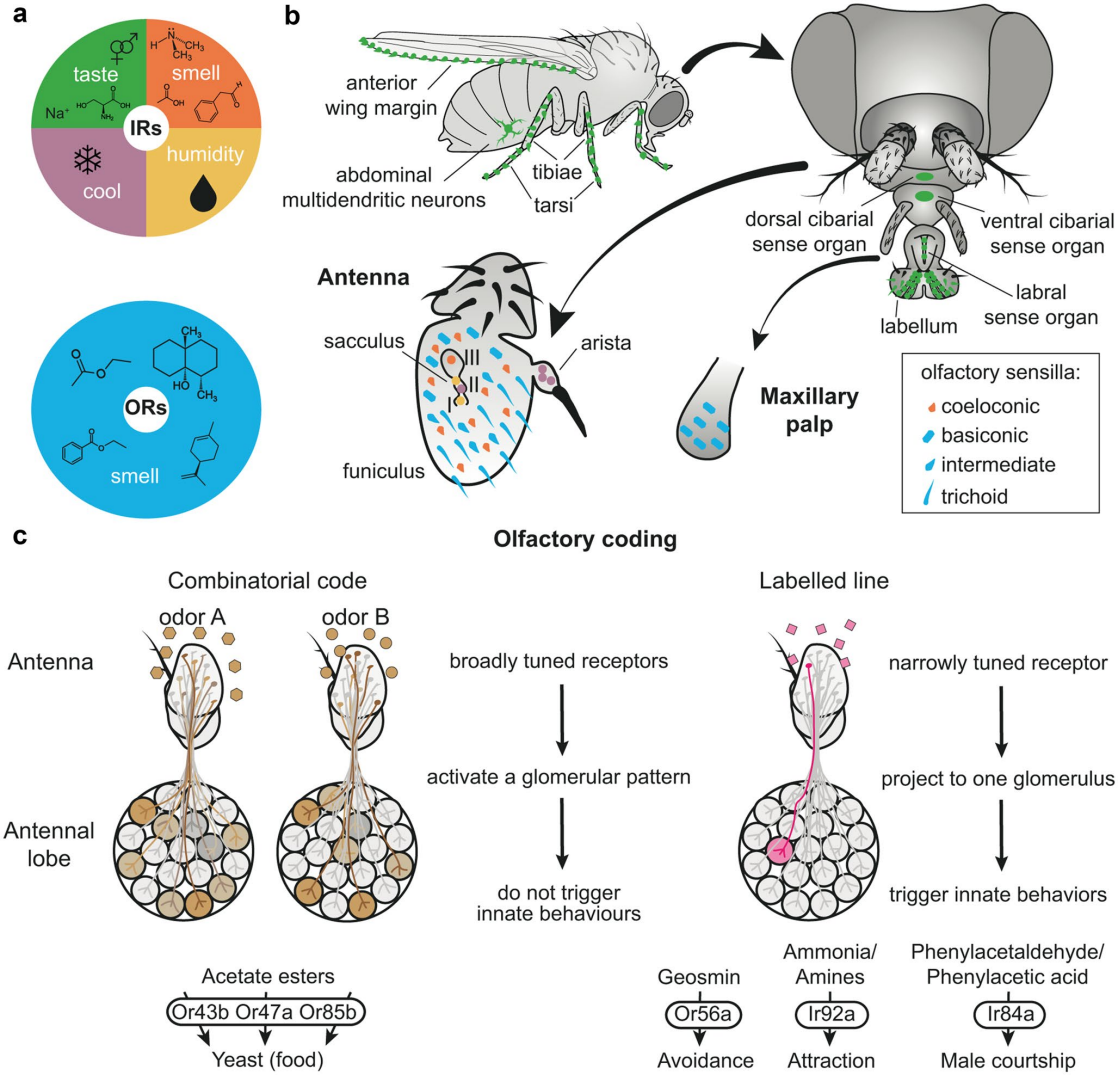
Functional roles and coding strategies of insect IRs and ORs. **a** Sensory modalities of ionotropic receptors (IRs) and odorant receptors (ORs). IRs are a multi-modal gene family for the detection of multiple taste qualities, volatile acids and amines, and environmental stimuli as humidity and cooling temperatures (Rimal and Lee 2018). ORs instead are specialized for the detection of a wide variety of volatile and semi-volatile compounds. **b** Distribution pattern of IRs and ORs in the adult vinegar fly *Drosophila melanogaster*. Candidate IR taste and pheromone receptors are expressed in the anterior margin of the wing, on abdominal multidendritic neurons, on the tibiae and tarsi of the legs, and on the labellar, labral and cibarial sense organs (Koh et al. 2014; Sánchez-Alcañiz et al. 2018). The antennal funiculus and the maxillary palps are the two main olfactory organs and they are covered with porous sensilla. The coeloconic sensilla house olfactory neurons expressing mainly IRs, while the basiconic, intermediate, and trichoid sensilla house OR-expressing neurons. The antenna houses two other sensory structures, namely, the sacculus and the arista, that express IRs involved in humidity and cooling sensing (Frank et al. 2017; Knecht et al. 2017). **c** IRs and ORs allow insects to detect a wide range of ecologically relevant chemicals through a combinatorial code and a set of labeled lines. In a combinatorial code, each odor is detected by multiple broadly tuned receptors and elicits a unique activation pattern of the antennal lobe glomeruli. Such mechanism allows the fly to exploit a wide array of food sources through the detection of multiple acetate esters produced by yeasts (Mansourian and Stensmyr 2015). Odors detected through labeled lines activate only one tuning receptor and trigger specific innate behaviors (Grosjean et al. 2011; Min et al. 2013; Stensmyr et al. 2012)

While ORs solely detect volatile chemosignals, IRs are multimodal receptive entities (Fig. 1a). In addition to their olfactory role, IRs are involved in taste sensation, in hygrosensation, and in cool temperature sensation thereby, for example, synchronizing circadian rhythms (Rimal and Lee 2018). The olfactory receptors are localized in the olfactory sensory organs, the antennae, and the maxillary palps (Fig. 1b). These organs are covered with sensilla, i.e., cuticular, hair-like structures that house the receptor expressing dendrites of olfactory sensory neurons (OSNs). Each sensillum contains one to four OSNs that express different receptors. The odor molecules pass into the sensilla through pores and diffuse to the dendrites, facilitated by odor binding proteins (OBPs), especially for hydrophobic odor molecules. The sensillum lymph contains K^+^ at an unusually high concentration (Kaissling and Thorson 1980). Sensilla are classified according to their shape in coeloconic, basiconic, intermediate, and trichoid (Fig. 1b). These sensilla types differ in localization and in the OSN types they contain. IR expressing cells occur in coeloconic sensilla, while OR expressing OSNs appear in basiconic, intermediate, and in trichoid sensilla.

The olfactory system has to detect and to identify a large number of different volatile signal molecules that belong to such diverse chemical classes as acids, alcohols, esters, or aromatics (Keller and Vosshall 2016). Additional complexity arises from the presentation of odors as plumes containing many individual odorants. Given these challenging tasks, the olfactory system has to be equipped with a sufficient number of sensory units and it has to allow an efficient processing of input information. The resolution of chemoreceptors is limited by the noise due to Brownian motion of signaling molecules. First investigated in bacterial chemotaxis, the accuracy of concentration detection were limited when only a few molecules occupied the space of a cell volume (Berg and Purcell 1977). In such a case, the fractional accuracy of the detected chemosignal concentration is inversely related to its diffusion coefficient, the cell radius, and the detection time. The resolution can thus be enhanced by increasing the receptive area or by prolonging the detection time (Bialek and Setayeshgar 2005). The sometimes impressive size of insect antenna may apply the principle to improve odor detection by enhancing the detector area. The other way, to improve the resolution by prolonged detection time is limited during insect flight as they have to detect odors present at varying concentrations as fast as possible.

For an appropriate processing of olfactory signals, two main strategies have been developed (Fig. 1c). The first one is combinatorial coding; the second one is based on so-called labeled lines (Galizia 2014; Grabe and Sachse 2018; Haverkamp et al. 2018). For a combinatorial coding IRs and ORs are broadly tuned; i.e., a range of different odors activate one receptor type. Alternatively, one specific odor can activate various receptor types. The odor identity is coded by the subpopulation of receptor types it activates. By contrast, in labeled lines the receptor types are narrowly tuned and detect especially important compounds such as pheromones or ecologically relevant odors. For example, in *Drosophila melanogaster*, the Or56a solely detects geosmin, an odor released by toxin-producing molds, and the OR activation immediately triggers an avoidance response (Stensmyr et al. 2012). Furthermore, *Drosophila* OSNs expressing Ir92a mediate attraction towards ammonia and amines (Min et al. 2013). As another example, activation of Ir84a by food-derived odors triggers male fly courtship behavior (Grosjean et al. 2011) (Fig. 1c). The internal representation of the outside world provided by the sensory machinery and the subsequent processing by the nervous system constitutes the basis for behavioral decisions. The success of behavioral responses relies on the quality of this representation. While a very precise representation may be metabolically costly and an inappropriate representation can cause wrong decisions, the choice of an appropriate quality level is the subject of an optimizing process (Mlynarski and Hermundstad 2018).

## Genetic basis and evolution

**Fig. 2:**
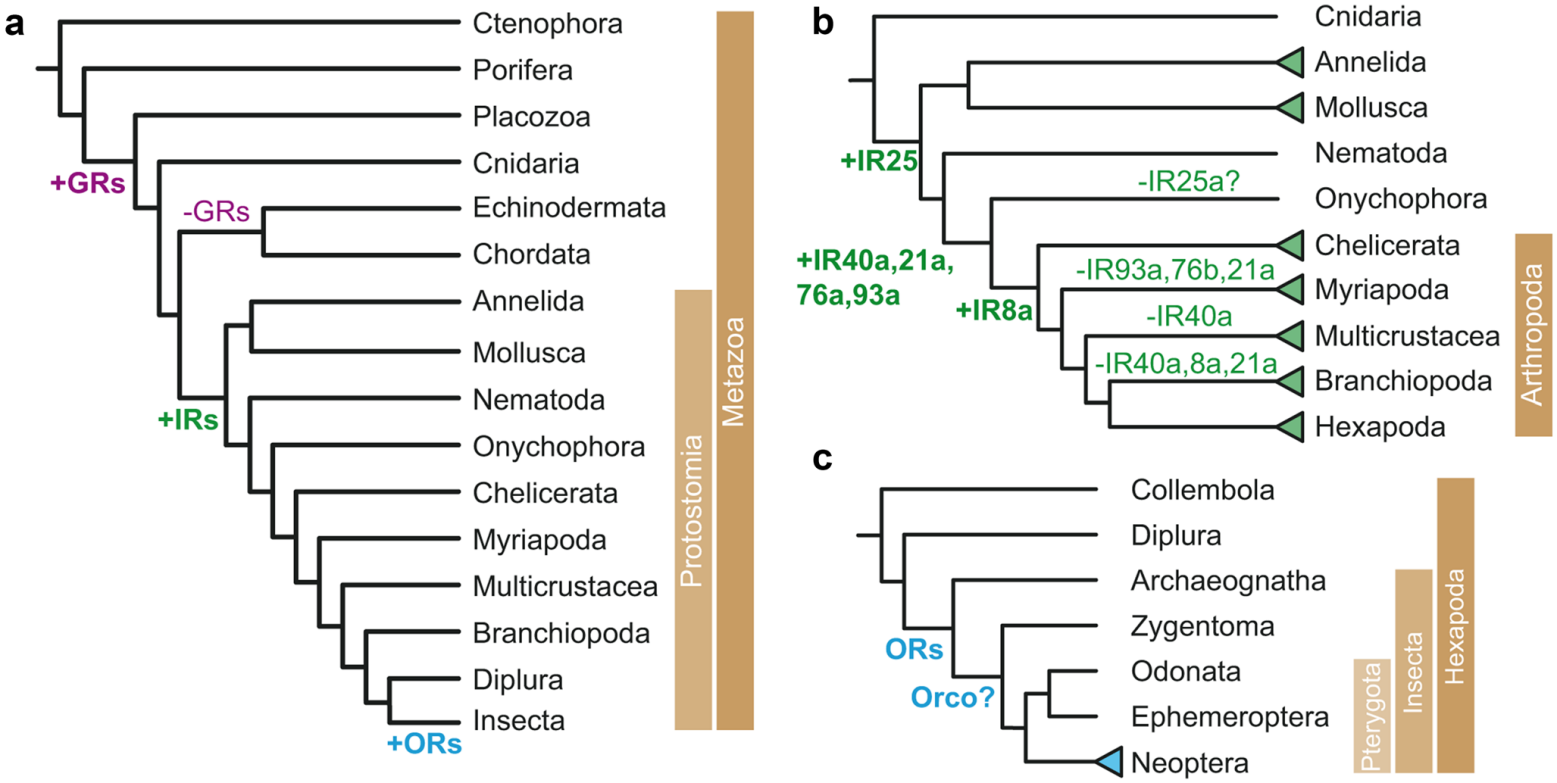
Evolution of arthropod ionotropic and odorant receptors. **a** Emergence of the three main classes of arthropod chemoreceptors: gustatory receptors (GRs), ionotropic receptors (IRs), and odorant receptors (ORs). GRs arose early in the evolution of Metazoa. While being lost in Deuterostomia (Chordata and Echinodermata), they represent the most ancient arthropod chemosensory receptor class between the three. IRs emerged with Protostomia (Croset et al. 2010), while ORs evolved from GRs and they represent the most recent chemosensory class (Robertson et al. 2003). **b** Conserved antennal IR subfamily gain dynamics. Based on their expression pattern, IRs can be divided in conserved “antennal” IRs and species-specific “divergent” IRs (Croset et al. 2010). Gain of IR gene subfamilies is shown as green triangles. The gain and loss dynamics for the six arthropod antennal IR subfamilies is highlighted in green. Recent results show that none of these families is unique to insects (Eyun et al. 2017). **c** Evolution of insect ORs. The emergence of ORs pre-dates the evolution of winged insects (Pterygota) and OR genes have been detected in basal insects. The conserved olfactory receptor-coreceptor (Orco) has not been detected in Archaeognatha and may be evolved later or being lost in this Order (Brand et al. 2018; Thoma et al. 2019). Winged insects show a huge expansion in their number of OR genes (blue triangle)

There are remarkable similarities between neuronal synapses and chemosensory systems with regard to structures, mechanisms, and expressed molecules (Shaham 2010). At the receptive region, i.e., the postsynapse or the dendrite of a chemosensory cell, respectively, the signaling molecules bind to their specific receptors. An important subpopulation in mammalian central synapses is the ionotropic glutamate receptors. Insect olfactory receptors belonging to the IR family are structurally related to these glutamatergic receptors (Benton et al. 2009). A comparative genomic analysis revealed that IR proteins occur in all Protostomia but not outside (Fig. 2a). The IRs form subfamilies displaying a pronounced gain and loss dynamic as illustrated in Fig. 2b. The IRs form heterotetramers that contain variable, odor-specific IRX proteins, and co-receptor IRcoY proteins. In *Drosophila*, for example, co-receptor proteins are Ir8a and IR25a. As the co-receptor proteins show an extended amino terminal domain (ATD) as well as a ligand-binding domain (LBD), there is a quite pronounced similarity to iGluRs. In addition, the tetrameric IR complexes can contain up to three different subunits. An example for an IR tetramer built up by dimerisation of dimers was seen in *Drosophila* IR84a and IR8a (Abuin et al. 2011).

Regarding the functional principle, chemoreceptors operate as ionotropic or as metabotropic receptors (Wicher and Große-Wilde 2017). The actual choice depends on the properties required for the task. For fast chemosignal processing, ionotropic receptors were preferred, whereas the signal amplification by intracellular cascades are advantageous for a weak signal strength. As complement to the ligand-gated IRs, many chemoreceptive proteins belong to the G protein-coupled receptor (GPCR) family (Bargmann 2006). Insect OR proteins share the 7-transmembrane domain topology but they differ from GPCRs in various aspects. They are inversely oriented within the membrane as the N-terminus of OR proteins is intracellular and the C-terminus is extracellular (Benton et al. 2006; Lundin et al. 2007). Furthermore, OR proteins have no sequence similarity with GPCRs (Nordström et al. 2011). Considering structure and function, ORs are related to gustatory receptors (GRs), and they constitute an expanded lineage within the GR superfamily (Robertson et al. 2003). While GRs occur in all arthropods sequenced so far, the ORs are restricted to insects. Genes related to the GRs were found as gustatory related (GUR) genes in the nematode *C. elegans* and across the Protostomia as gustatory receptor-like (GRL) genes (Benton 2015; Robertson 2015). In the plant *A. thaliana* there are six proteins that contain a domain of unknown function (DUF3537) which shares some characteristics with GR, GUR, and GRL proteins, such as sequence similarity, heptahelical structure, and orientation within the plasma membrane (Benton 2015). Whereas the origin of the GR family goes back to the evolution of animals, the GRs disappeared in vertebrates (Benton 2015; Robertson 2015). (Fig. 2a).

The evolution of ORs may have happened in parallel with that of terrestrial insects from their aquatic ancestors (Brand et al. 2018; Robertson et al. 2003). Regarding functionality, this corresponds to the transition from detecting dissolved to volatile chemosignals. While OR genes were absent in non-insect hexapods such as springtails, they were found in winged and non-winged insects ( Brand et al. 2018). According to a phylogenetic analysis of OR genes there is an intermediate clade of primitive ORs expressed in bristletails, firebrats, or silverfish between GRs and the ORs of flying insects (Thoma et al. 2019). These primitive ORs then disappeared in flying insects, e.g., in damselflies and dragonflies. Furthermore, in winged insects there was a massive expansion of OR genes (Missbach et al. 2014; Thoma et al. 2019). These ORs are heteromeric constructs containing variable, odor-specific OrX proteins, and highly conserved co-receptor proteins (Orco) (Larsson, et al. 2004; Neuhaus et al. 2005; Soffan et al. 2018). Compared with the more ancient IRs, the ORs are more sensitive and are capable of resolving fast changing odor pulses (Getahun et al. 2012). Furthermore, these ORs are tunable as their sensitivity can be regulated depending on previous odor stimuli (Getahun et al. 2013). Taken together, these properties improve the performance of the olfactory system in a situation-dependent manner. This may constitute a functional adaptation to the challenge flying insects that are exposed in that they need to detect and to follow faint filaments of odor plumes.

The current state of knowledge supports the view that the ORs evolved from the GRs in a stepwise process. Starting with primitive ORs in early terrestrial insects (Thoma et al. 2019), the olfactory system has expanded its receptor repertoire with the occurrence of Orco proteins in Zygentoma (Brand et al. 2018) and ended up with the versatile OR complexes in flying insects (Brand et al. 2018; Thoma et al. 2019) (Fig. 2c). For more detailed genomic information on olfactory receptors see the two recent reviews (Gomez-Diaz et al. 2018; Fleischer et al. 2018). 

## Structure and function

**Fig. 3:**
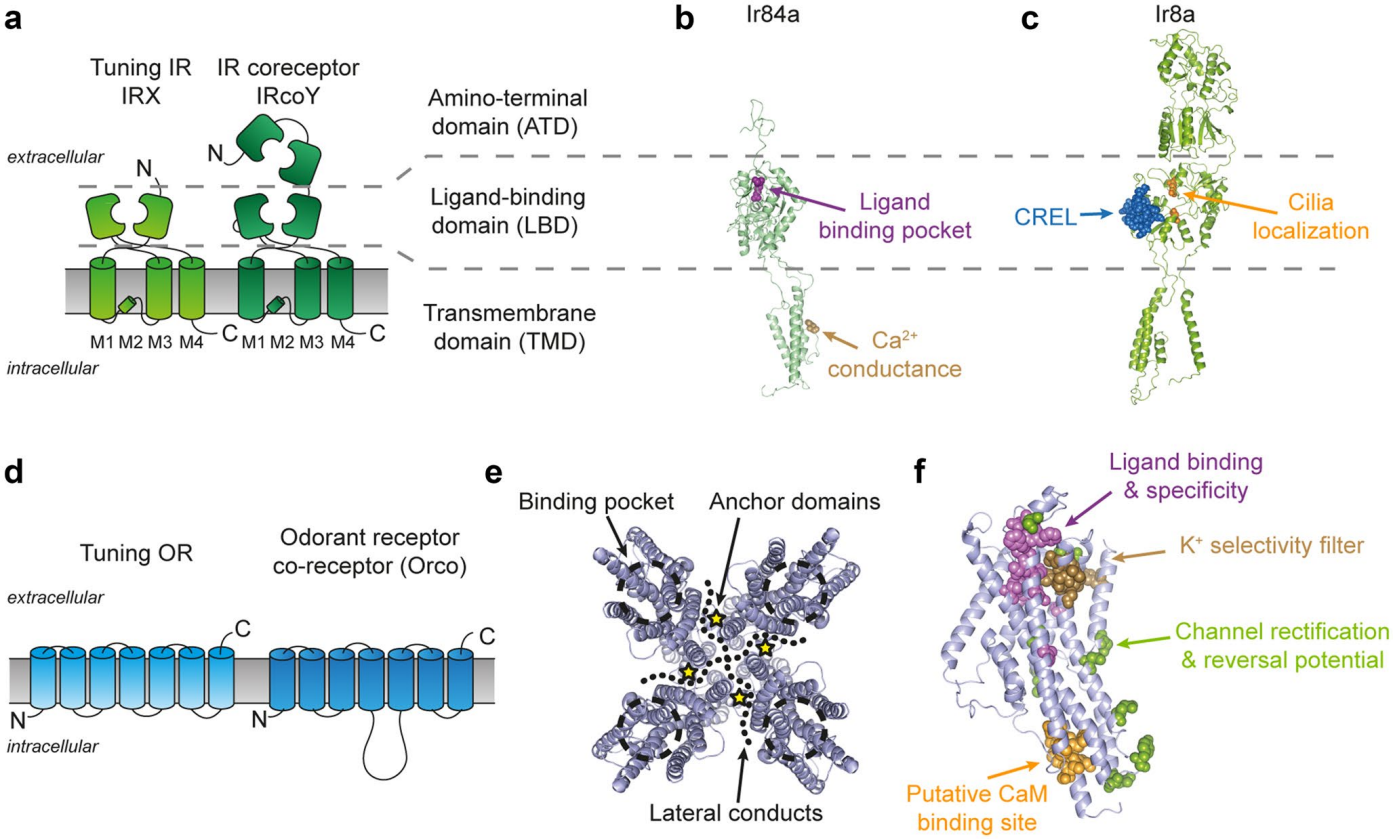
Structure-function of insect IRs and ORs. **a** Schematic representation of insect IRs. The functional unit of IRs is considered to be a heterotetramer made of two tuning receptor (IRX) and two coreceptor subunits (IRcoY) (Abuin et al. 2011; Abuin et al. 2019). The transmembrane domain (TMD) of both tuning receptor and coreceptor consists of four helices (M1-4). In the closely related AMPA ionotropic glutamate receptor (Croset et al. 2010), the re-entrant portion of the M2 loops forms the ion selectivity filter (Twomey et al. 2017). Both the tuning and coreceptor subunits possess the extracellular ligand-binding domain (LBD), but only the two coreceptors (i.e., Ir25a and Ir8a) possess an amino-terminal domain (ATD) (Croset et al. 2010). **b**–**c** Ribbon representation of a tuning (Ir84a) and a coreceptor (Ir8a) IR and characterized function of selected amino acid residues. **b** A glutamine in position 401 (in brown) in the M2 region of Ir84a is responsible for the Ca^2+^-dependent conductance of the Ir84a/Ir8a channels (Abuin et al. 2011). The LBD of tuning subunits houses the amino acid residues that form the ligand binding pocket and define the response specificity (Prieto-Godino et al. 2016; Prieto-Godino et al. 2017). In particular, for Ir84a see (Abuin et al. 2011; Cicconardi et al. 2017). **c** The LBD of the Ir8a coreceptor instead houses residues involved in the trafficking and correct cellular localization of the IR heteromers such as the coreceptor extracellular loop (CREL, in blue) (Abuin et al. 2019) and residues affecting IR localization to the sensory cilia (in orange) (Abuin et al. 2011). The coreceptor ATD also plays a major role in protein folding, heteromeric protein assembly, and/or cilia targeting (Abuin et al. 2011). Ir8a and Ir84a homology models were created in SWISS-MODEL (Waterhouse et al. 2018) with the *R. norvegicus* GluA2 structure (PDB: 6DLZ) (Twomey et al. 2018) as template following (Abuin et al. 2019). **d** Schematic representation of the 7 transmembrane-domain insect ORs. The functional unit of ORs is formed by heteromers made of a tuning OR and a coreceptor named Orco. **e** Top view ribbon representation of the tetrameric cryo-EM structure of Orco from the parasitic wasp *Apocrypta bakeri* (Butterwick et al. 2018). Highlighted are the binding pocket (dashed cicles), the anchor domain regions (star symbols), and the lateral conducts stemming from of the channel pore (dotted lines). **f** Functionally relevant residues of insect ORs, identified through mutagenesis studies, mapped on a *Drosophila melanogaster* Orco monomer. Critical residues are involved in the ligand binding and selectivity of ORs (in violet) (Corcoran et al. 2018; Hopf et al. 2015), a putative calmodulin binding site with modulatory function (Mukunda et al. 2014), K^+^ selectivity filter (in brown) (Wicher et al. 2008), and ion channel function (in green) (Hopf et al. 2015). The ribbon representation of *D. melanogaster* Orco was modeled on the *A. bakeri* cryo-EM structure using the I-TASSER server (Yang and Zhang 2015) and optimized using FoldX (Schymkowitz et al. 2005)

Similar to the related iGluRs, insect IRs are ligand-gated ion channels with odor molecules as ligands. They constitute nonselective cation channels passing Na^+^ and K^+^; some of them also Ca^2+^ (Abuin et al. 2011; Rytz et al. 2013). Within the IRX/IRcoY complexes of the odor specificity is determined by the tuning IRX partner (Abuin et al. 2011) (Fig. 3b). This is not so obvious as for the ORs as outlined below as there is also a LBD in IRcoY (Fig. 3a). But the LBD of co-receptors may serve other purposes than odor binding as shown for IR8a where the LBD is responsible for traffic and correct dendritic localization (Abuin et al. 2011; Abuin et al. 2019) (Fig. 3c). Odors may enhance the OSN activity, but they can also reduce it. Odor specificity for IRs and ORs is in general complementary, yet there is some overlap (Silbering et al. 2011). IR expressing OSNs need longer or stronger stimuli to become activated than OR-expressing neurons. On the other hand, IR-expressing neurons adapt more slowly (Getahun et al. 2012). And, also in contrast to ORs, IRs cannot be sensitized by repeated subthreshold odor stimuli (Getahun et al. 2013).

In winged insects the ORs are heteromeric complexes of heptahelical odor-specific OrX proteins (tuning OR) and the co-receptor protein Orco (German et al. 2013; Larsson et al. 2004; Neuhaus et al. 2005) (Fig. 3d). The OrX show a high degree of variability which usually reflects the ecological niche of a given species. By contrast, Orco proteins are quite conserved among species and they form a separate clade within OR proteins (Soffan et al. 2018). ORs represent ligand-gated ion channels (Sato et al. 2008; Wicher et al. 2008). They are non-selective cation channels that pass Na^+^, K^+^, and Ca^2+^. In the heterologous expression system Orco proteins also associate as non-selective cation channels, yet these channels are insensitive to odors. Instead, Orco channels are activated by cyclic nucleotides (Wicher et al. 2008) or synthetic agonists such as VUAA1 (Jones et al. 2011). While we still lack information on the subunit structure of OR channels, an Orco channel composition was recently elucidated by cryo-electron microscopy (cryo-EM) for the wasp *Apocrypta bakeri* (Butterwick et al. 2018). The central channel pore is formed by four Orco proteins and lined by the membrane spanning part of helix 7 (Fig. 3e). Near the cytoplasmic terminus the helices 5, 6, and 7 constitute four lateral conducting pathways as shown in Fig. 3e. The Orco channel can be activated by ligands such as the synthetic agonist VUAA1 by binding to an extracellular pocket formed by helices 1 to 6 (Fig. 3e). The Orco proteins bear cytoplasmic anchor domains that contribute to channel forming protein interaction (Fig. 3e). The selectivity filter is localized at the narrowest pore region near the extracellular face (Fig. 3f). Based on this Orco structure one might conclude that ORs are tetrameric assemblies of Orco and OrX subunits in which both proteins line up the pore. Such assumption gets support from the observation that specific channel properties rely on the specific OR composition (Pask et al. 2011). Composition with different OrX proteins leads to differences in the permeability to mono and divalent cations of the ORs. In addition, the effect of mutations in OrX and Orco proteins suggests that both protein types form the OR pore (Nakagawa et al. 2012).

While generally operating as odorant-gated channels, at least some ORs show a constitutive activity (Sato et al. 2008; Wicher et al. 2008). Such a background activity of cation channels confers pacemaker properties and thus determines the basal OSN activity. The response to an olfactory stimulus can be excitatory or inhibitory, and thus accelerate or slow down the OSN activity, respectively. Generation of an inhibitory response relies on a certain background activity of an OSN, and such activity requires a certain pacemaker activity. An odor-evoked inhibition can, for example, be obtained by inhibition of the constitutive OR activity (Cao et al. 2017).

The OSN background activity is determined by the OrX type expressed (Hallem et al. 2004). Moreover, OrX also determines whether the response to a given odor is excitatory or inhibitory (Hallem et al. 2004). It is also possible that one odor elicits an excitatory response, while another odor leads to an inhibitory response. The *Drosophila* Or59b, for example, is excited by ethyl acetate and inhibited by linalool (de Bruyne et al. 2001). A comprehensive analysis of *Drosophila* OR responses is given by Hallem and Carlson (2006). In general, ORs respond to more than one odor, and the responses may vary from narrowly tuned to broadly tuned.

Orco proteins occur both in somatic and in dendritic regions, but OrX proteins occur only in dendrites (Benton et al. 2006). The Orco proteins in the soma may form constitutively active channels acting as pacemakers that determine the resting activity of OSNs (Stengl and Funk 2013).

## Regulation

**Fig. 4:**
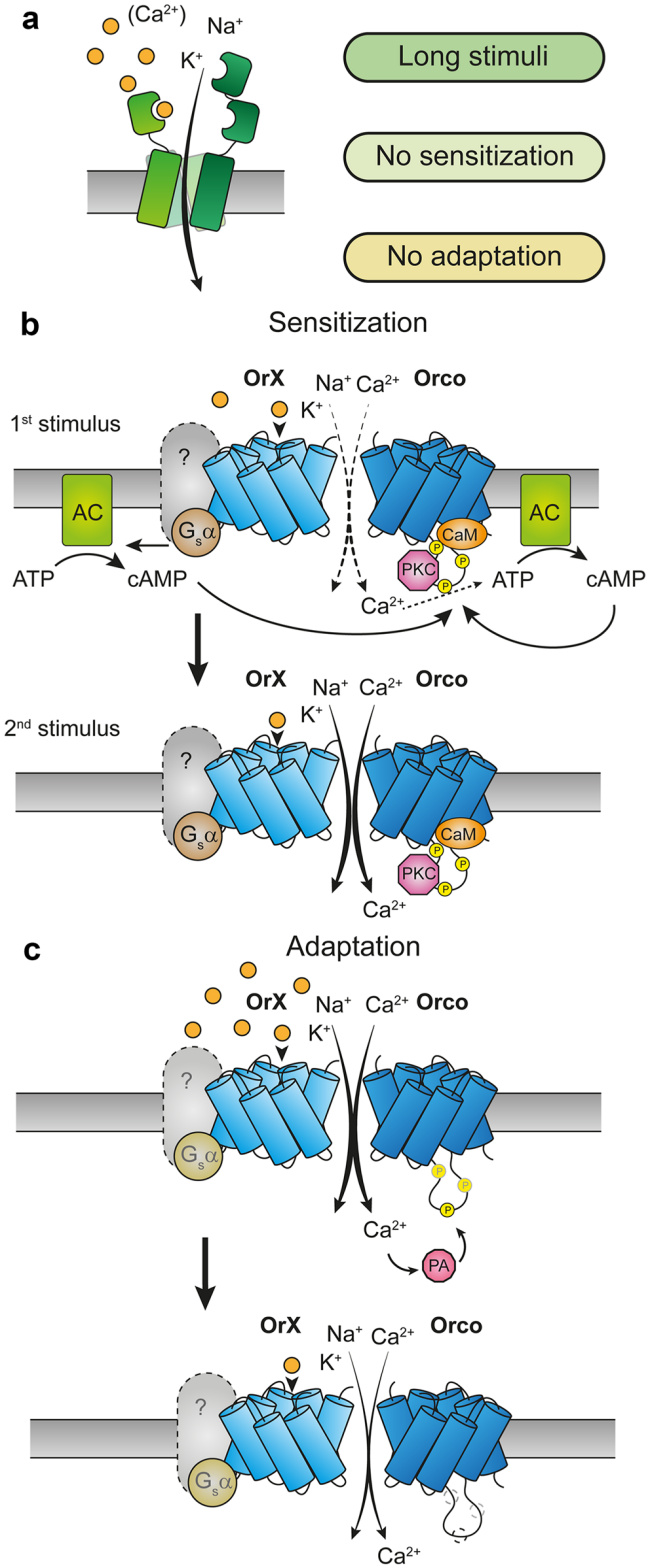
Function and regulation of IRs and ORs. **a** IRs are ligand-gated ion channels mainly permeable to monovalent cations (Na^+^, K^+^). Ca^2+^ conductance in, e.g., Ir84a depends on the Q401 residue in the channel M2 region described in Fig. 3. IR channels are activated by long-lasting stimuli, and their activity is not modulated by sensitization or adaptation mechanisms (Getahun et al. 2012). **b**–**c** ORs are nonselective cation channels modulated by intracellular signaling cascades. Activation of these cascades depends on the stimulus strength and may lead to receptor sensitization (**b**) or adaptation (**c**). **b** Activation of a tuning OrX can produce an increase in intracellular cAMP levels in a Ca^2+^-dependent and Ca^2+^-independent manner, leading to the activation of adenylyl cyclases (AC) (Miazzi et al. 2016). Upon phosphorylation of Orco via protein kinase C (PKC), cAMP can increase the activity of OR channels (bold arrow) when repeatedly exposed to sub-threshold stimuli (Getahun et al. 2013; Getahun et al. 2016; Sargsyan et al. 2011; Wicher et al. 2008). Receptor sensitization requires also calmodulin (CaM), as this process can be suppressed via CaM pharmacological inhibition (Mukunda et al. 2016). **c** A single Orco residue, S289, governs OR adaptation and desensitization. When Ca^2+^-dependent phosphatases (PA) dephosphorylate Orco at position S298, the sensitivity of ORs to their agonist is diminished (light arrow) (Guo et al. 2017; Guo and Smith 2017)

Most players in biological systems are subject to regulative processes that adjust their functional properties according to the actual requirements. In special, the olfactory system needs to detect volatile chemical signals presented at enormously differing concentrations. In a big distance from the source, chemosignals are faint and dispersed, as for example sex pheromones released by a potential mating partner. On the other hand, the signal can be very strong, e.g., near a flowering plant. As mentioned in the previous paragraph, IRs need stronger stimuli and they show less adaptation in comparison with ORs. With these properties the IRs are olfactory receptors suitable to detect messages of considerable strength or near their source (Fig. 4a). By contrast, ORs respond to weak stimuli, but they also show fast adaptation (Nagel and Wilson 2011; Getahun et al. 2012).

Various intracellular signaling cascades regulate the OR performance (Fleischer et al. 2018; Wicher 2018). Though insect ORs are not GPCRs, the OR function in a heterologous expression system was characterized by means of its interaction with G proteins (Wetzel et al. 2001). In addition, the expression of G proteins in the insect antenna has been demonstrated (Boto et al. 2010) and an appropriate odor detection was found to rely on G protein function (Deng et al. 2011; Ignatious Raja et al. 2014; Kain et al. 2008). In line with this, the olfactory processing was found to be affected by manipulations within the cAMP cascade downstream stimulatory G proteins. A reduction of cAMP levels due to overexpression of the phosphodiesterase *dunce* or by adenylyl cyclase inhibition reduced olfactory responses (Getahun et al. 2013; Gomez-Diaz et al. 2004), while elevating the cAMP level lowered the detection threshold for an odor (Getahun et al. 2013) as well as increased the resting activity of OSNs (Deng et al. 2011). Also, the signaling cascade downstream G_q_ proteins play a role in olfactory processing (Krieger and Breer 1999). Impaired odor responses, for example, were observed in *norpA* and *plc21* mutants lacking phospholipase C function (Kain et al. 2008; Riesgo-Escovar et al. 1995). Interestingly, the pheromone signal transduction process in the hawkmoth *Manduca sexta* operates exclusively via metabotropic pathways (Nolte et al. 2016; Stengl 2010).

The sensitivity of various ORs, e.g., food odor detecting receptors, can change in response to the strength of stimuli. For example, repetitive near-threshold stimuli enhance their sensitivity (Getahun et al. 2013). A key role in this sensitization process is played by Orco proteins (Fig. 4b). Orco channels are activated by cAMP (Wicher et al. 2008), but this requires a certain level of Orco phosphorylation by protein kinase C (PKC) (Sargsyan et al. 2011). Interestingly, the cAMP production is stimulated by odor induced OR activation (Miazzi et al. 2016). As Orco forms non-selective cation channels passing also Ca^2+^, Orco activation is accompanied by Ca^2+^ influx which activates calmodulin (CaM), stimulates CaM binding to Orco, and in turn enhances Orco channel activity (Mukunda et al. 2014). The CaM contribution is necessary for the sensitization process as pharmacological inhibition or mutation within the Orco CaM binding site abolishes the sensitization effect (Mukunda et al. 2016). There is another putative feedback loop in Orco function which involves PKC activation by Ca^2+^ and also elevates the current flow through Orco channels (Getahun et al. 2016; Sargsyan et al. 2011). These autoregulative processes lead to OR sensitization and cause larger ion fluxes (Fig. 4b).

The functional state of receptors is tuned according to strength and duration of their activation. For example, long-lasting or repetitively presented stimuli cause adaptation of the OR response as described by the Weber-Fechner law with logarithmic relationship between stimulus and perception (Cao et al. 2016; Nagel and Wilson 2011). An important parameter accounting for an adaptation is the cytoplasmic Ca^2+^ level which rises when OR channels are open (Cao et al. 2016). Prolonged odor stimuli lead to OR desensitization. In *Drosophila*, the Orco S289 residue is one of the PKC phosphorylation sites controlling Orco function (Sargsyan et al. 2011), and it becomes dephosphorylated under such circumstances. This reduces the sensitivity of the active OR to the permanently presented odor (Guo et al. 2017). Responsible for the Orco dephosphorylation is probably a Ca^2+^-activated phosphatase (Guo and Smith 2017) (Fig. 4c). An alternative way to regulate olfactory responses is the control of receptor protein expression. In *Drosophila*, the hedgehog pathway controls the transfer of OR proteins into the dendritic membrane (Sanchez et al. 2016). As hedgehog is expressed in the OSNs, this represents an autoregulatory process.

## Other players in the peripheral odor response

**Fig. 5:**
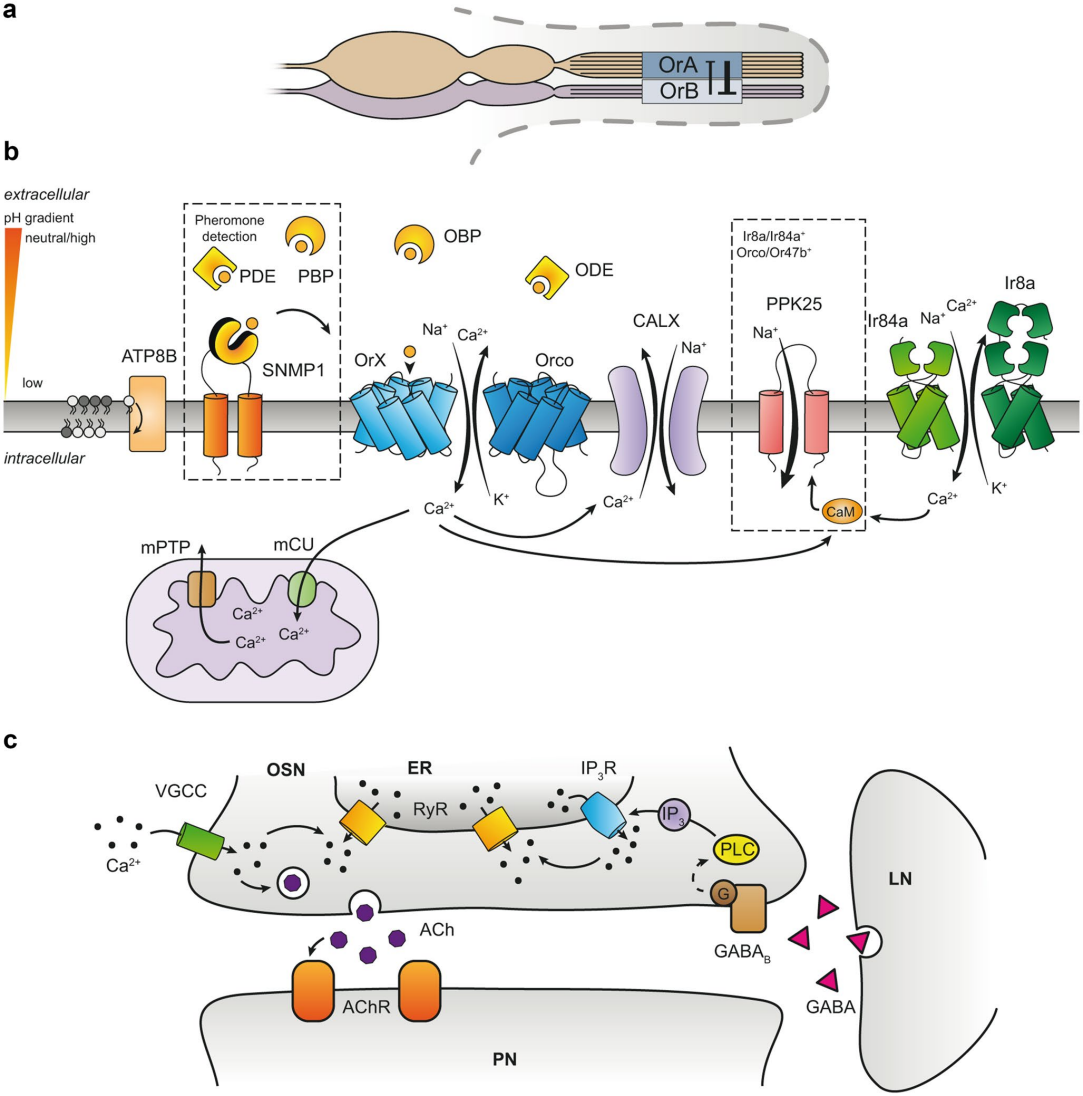
Additional players in the modulation of insect olfactory receptors response. Several mechanisms add layers of complexity to the modulation of insect olfactory receptors. **a** Olfactory sensory neurons (OSNs) housed within the same sensillum can differ in their morphology and influence each other’s activity by means of ephaptic interactions (Zhang et al. 2019). **b** Modulation of olfactory receptors’ activity at the interface between the extracellular sensillum lymph and the cytoplasm. OR-ligand interactions are influenced by odor- and pheromone-binding proteins (OBPs and PBPs) and odor- or pheromone-degradating enzymes (ODEs, PDEs) (Larter et al. 2016; Leal 2013; Xiao et al. 2019). In addition, lipid-derived pheromones can require additional membrane proteins for efficient detection, such as SNMP1 (Gomez-Diaz et al. 2016). Activation of ORs and a subset of IRs, e.g., IR84a, increases the intracellular Ca^2+^ concentration ([Ca^2+^]_i_). In Or47b- or Ir84a-expressing OSNs this [Ca^2+^]_i_ increase can sensitize the OSN in a CaM-dependent way through the DEG/ENaC channel PPK25 (Ng et al. 2019). Cytoplasmic Ca^2+^ can be sequestered in the mitochondria through the mitochondrial calcium uniporter (mCU), while Ca^2+^ can be release from this organelle through the mitochondrial permeability transition pore (mPTP) (Lucke et al. 2020). Moreover, cytoplasmic Ca^2+^ can be extruded through the Na^+^/Ca^2+^ exchanger (CALX) (Halty-deLeon et al. 2018). OR function and/or intracellular trafficking is also affected by other proteins, such as the ATP8B flippase, that is involved in maintaining the phospholipid asymmetry of the plasma membrane (Ha et al. 2014; Liu et al. 2014). **c** The endoplasmic reticulum (ER) instead plays a role in the adaptation after long-lasting stimuli. After an odor response, the opening of voltage-gated Ca^2+^ channels (VGCCs) at the presynaptic terminus can lead to the opening of ryanodine receptors (RyRs) and trigger a Ca^2+^-induced Ca^2+^ release (CICR). The resulting release of acetylcholine (ACh) stimulates the projection neuron's (PN) ACh receptors (AChRs) and may also activate—directly or indirectly—the release of GABA from associated local interneurons (LN). GABA release can in turn activate the OSN inositol 1,4,5-triphosphate receptors (IP_3_Rs) via the phospholipase C (PLC) pathway activated by GABAB receptors. The resulting Ca^2+^ release from the ER can activate RyRs and lead to an additional amplification of the signal through CICR (Murmu et al. 2010, 2011)

The olfactory receptors are the first instance to detect and to process a chemosignal. However, they operate as a chain link within a broader cooperative system made of protein networks, organelles, and cellular interactions that influence how odors are perceived. The sensillum environment in which receptors are expressed strongly influences their activity. The relative size of OSNs sharing the same sensillum determines the degree of lateral inhibition that these OSNs are subjected to through ephaptic interactions (Zhang et al. 2019). The bigger neuron within a pair can exert a lateral inhibition on the smaller neuron by exercising a stronger influence on the extracellular potential at the shared sensillum lymph (Fig. 5a). Such effect allows a faster processing of odor mixtures at the periphery by favoring the information carried by the larger neuron (Zhang et al. 2019).

Olfactory receptors reside at the interface between the extracellular sensillum lymph and the intracellular environment. Their activity is influenced by many proteins that are present in these spaces (Fig. 5b). The sensillum lymph contains a variety of soluble proteins such as OBPs and pheromone-binding proteins (PBPs) and odor- and pheromone-degradating enzymes (ODEs and PDEs) that can influence the sensitivity of ORs and, in general, the availability of hydrophobic odor molecules in the extracellular aqueous environment (Leal 2013). The study of their function and modes of action is still an area of active investigation. The pH gradient between the lymph at the sensillum pores and the OSN plasma membrane at the sensory cilia is thought to play a major role in determining the binding of hydrophobic odor molecules to OBPs. By using such gradient, OBPs can bind to volatile molecules in close contact with the surface of the antenna and release them in proximity of ORs (Leal 2013). However, recent work showed that the deletion of the most abundant OBPs in six vinegar fly basiconic sensilla did not reduce the intensity of odor responses (Larter et al. 2016; Xiao et al. 2019). In addition, a large number of membrane proteins influence the function and regulation of insect olfactory receptors. The sensory neuron membrane protein 1 (SNMP1) is important for the detection of lipid-derived pheromones by facilitating their delivery to the binding pocket of their cognate OR (Benton et al. 2007; Gomez-Diaz et al. 2016; Rogers et al. 2001; Rogers et al. 1997). The phospholipid flippase ATP8B instead was found to be required for the correct trafficking of ORs to the sensory cilia and/or to influence the receptor sensitivity to odors (Ha et al. 2014; Liu et al. 2014). ORs and a subset of IRs, including Ir84a, are permeable to Ca^2+^ that can act as a second messenger and modulate the OSN response. In the vinegar fly, Or47b- and Ir84a-expressing OSNs also express Pickpocket 25 (PPK25) (Starostina et al. 2012). This DEG/ENaC channel is activated by Ca^2+^ through a CaM-dependent mechanism and is responsible for the age-dependent amplification of the response to aphrodisiac odors in male flies (Ng et al. 2019).

The dynamics of the intracellular calcium concentration ([Ca^2+^]_i_) after the activation of ORs is influenced by many players. Ca^2+^ can be sequestered by mitochondria through the mitochondrial calcium uniporter (mCU) and released through the mitochondrial permeability transition pore (mPTP) (Lucke et al. 2020). The resulting buffering effect of mitochondria on [Ca^2+^]_i_ contributes in shaping the late phase and the termination of the OR response (Lucke et al. 2020). The Na^+^/Ca^2+^ exchanger CALX also plays a role in defining the termination of the odor response, by extruding the intracellular Ca^2+^ to the sensillum lymph (Halty-deLeon et al. 2018). Another role for Ca^2+^ in the odor response termination is to trigger Ca^2+^-activated Cl^-^ channels (Pézier et al. 2010). Furthermore, Ca^2+^ can modulate the OSN response by acting as a second messenger even far away from the sensory cilia where the odor molecules are first detected. In the antennal lobe, GABAergic local interneurons (LNs) shape the OSN adaptation to long-lasting stimuli through a Ca^2+^ release from the endoplasmic reticulum via inositol 1,4,5-triphosphate (IP_3_R) and ryanodine (RyR) receptors (Murmu et al. 2010, 2011) (Fig. 5c). In addition, LN can adjust the gain of the OSN odor responses through inhibitory feedback mechanisms at the axon terminals (Olsen and Wilson 2008; Root et al. 2008).

## Outlook and application

In conclusion, even decades after their discovery the insect IRs and ORs are still subject of intense research. The recent advances in understanding structure (Abuin et al. 2019; Butterwick et al. 2018) and evolution (Brand et al. 2018; Eyun et al. 2017; Thoma et al. 2019) of these receptor families impressively illustrate the dynamic progress in that research area and provide new tools to extend our knowledge and to explore new directions. Nevertheless important information such as the structure of native OR complexes is still missing. Similarly, the strong odor signal amplification that allows male moths to detect only a few pheromone molecules remains to be understood in detail.

A remarkable application of insect odorant receptor proteins is their use in the fabrication of biosensors (Bohbot and Vernick 2020). Such bioelectric noses utilize the odor-specific OrX proteins imbedded in liposomes (Khadka et al. 2019) or nanodiscs (Murugathas et al. 2019) as sensing elements for food screening. Binding of the odors the OrX proteins are specific for leads to an electric response that does not require any Orco protein. The advantage of OR proteins as biosensing elements is the combination of ligand specificity with a high sensitivity.
